# Supplementary material for: Thrombospondin-1 Silencing Improves Lymphocyte Infiltration in Tumors and Response to Anti-PD-1 in Triple-Negative Breast Cancer
Source: Cancers (Basel). 2021 Aug 12;13(16):4059. doi: 10.3390/cancers13164059 (PMC8391594; doi:10.3390/cancers13164059)
Supplement: Supplementary file 1 [file cancers-13-04059-s001.zip › cancers-1301665-supplementary.pdf]

A/ Epithelial Mesenchymal Transition

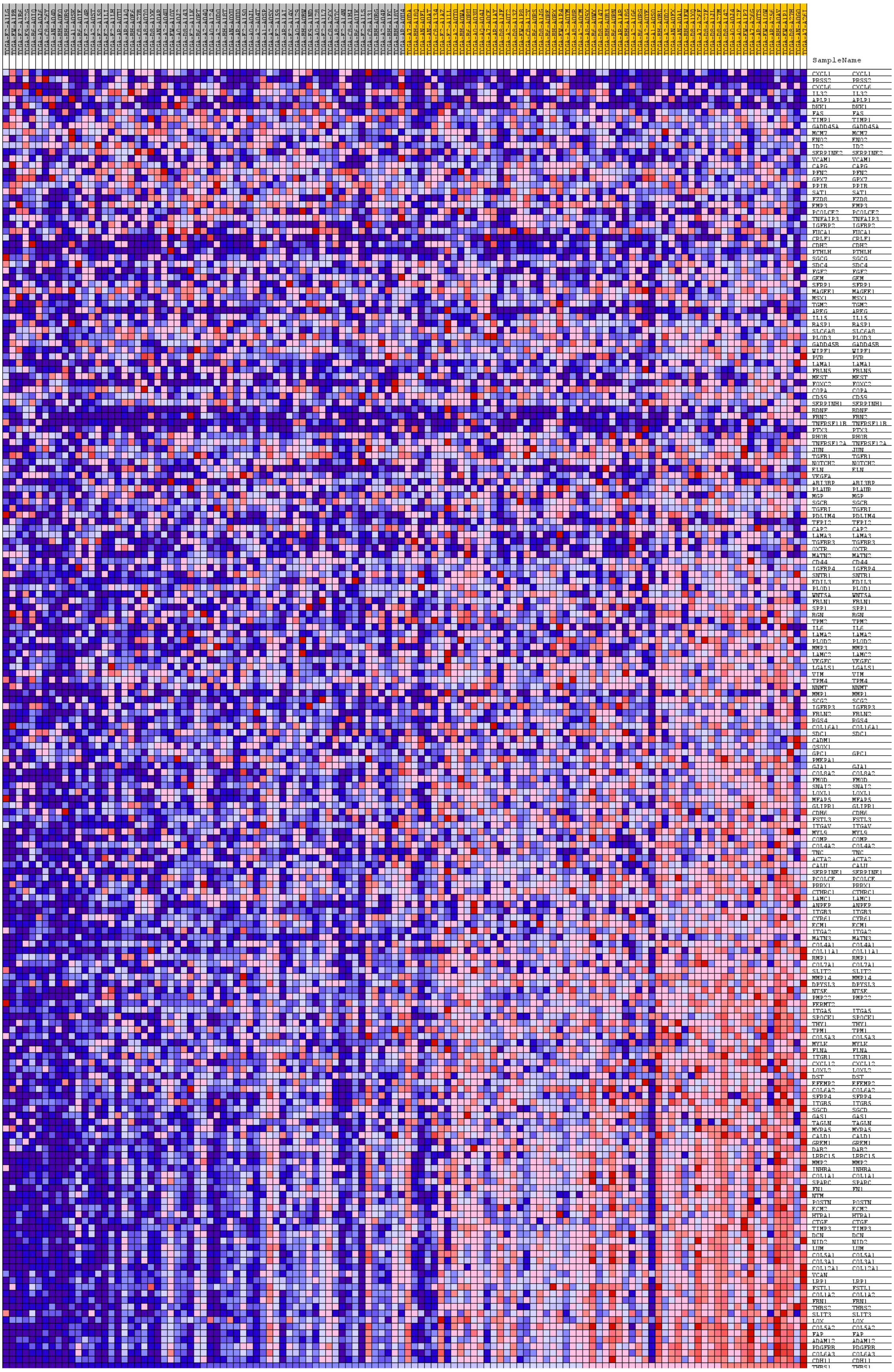

B/ TGF Beta Signaling

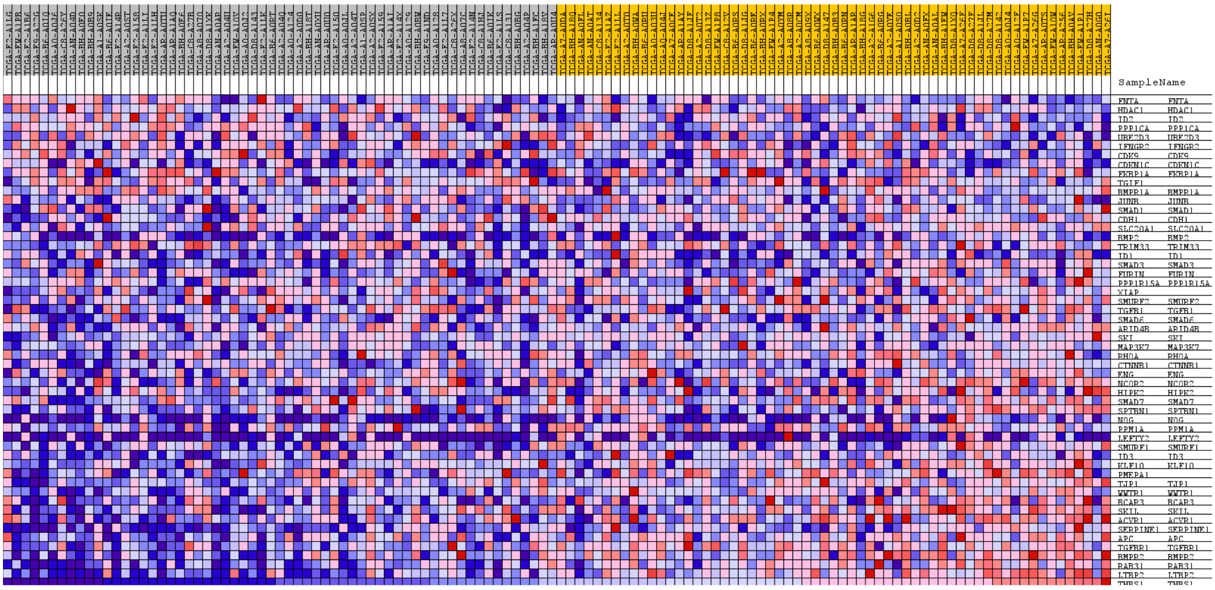

C/ Lymphocyte activation

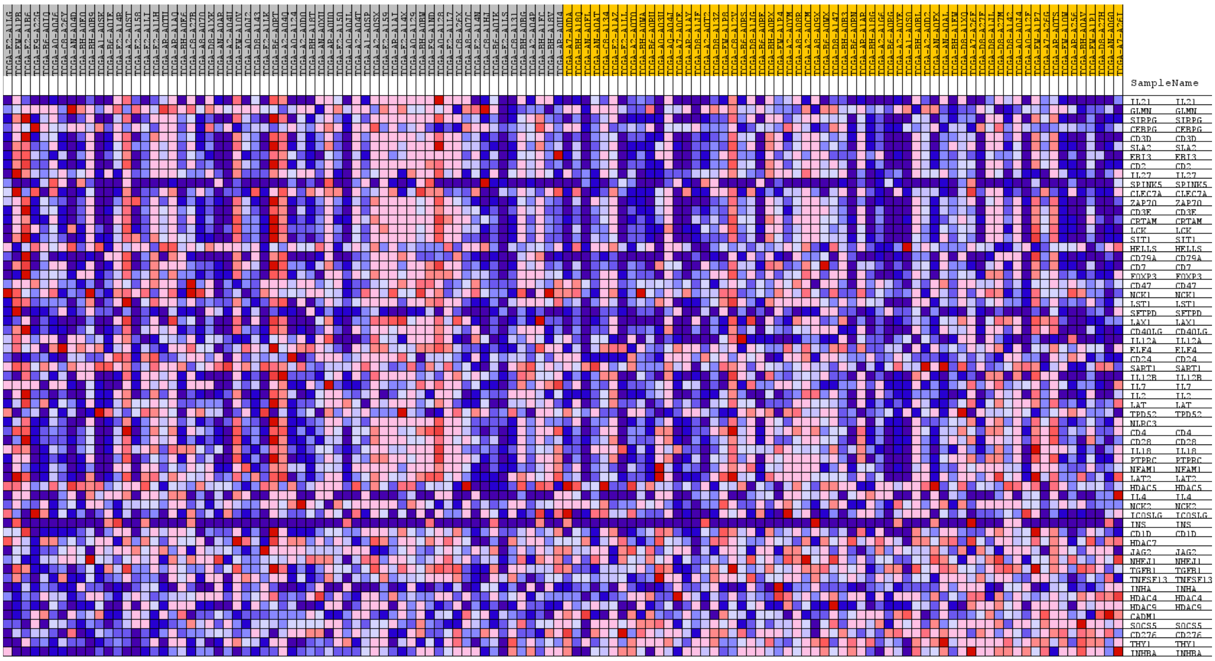

D/ Interferon Gamma Response

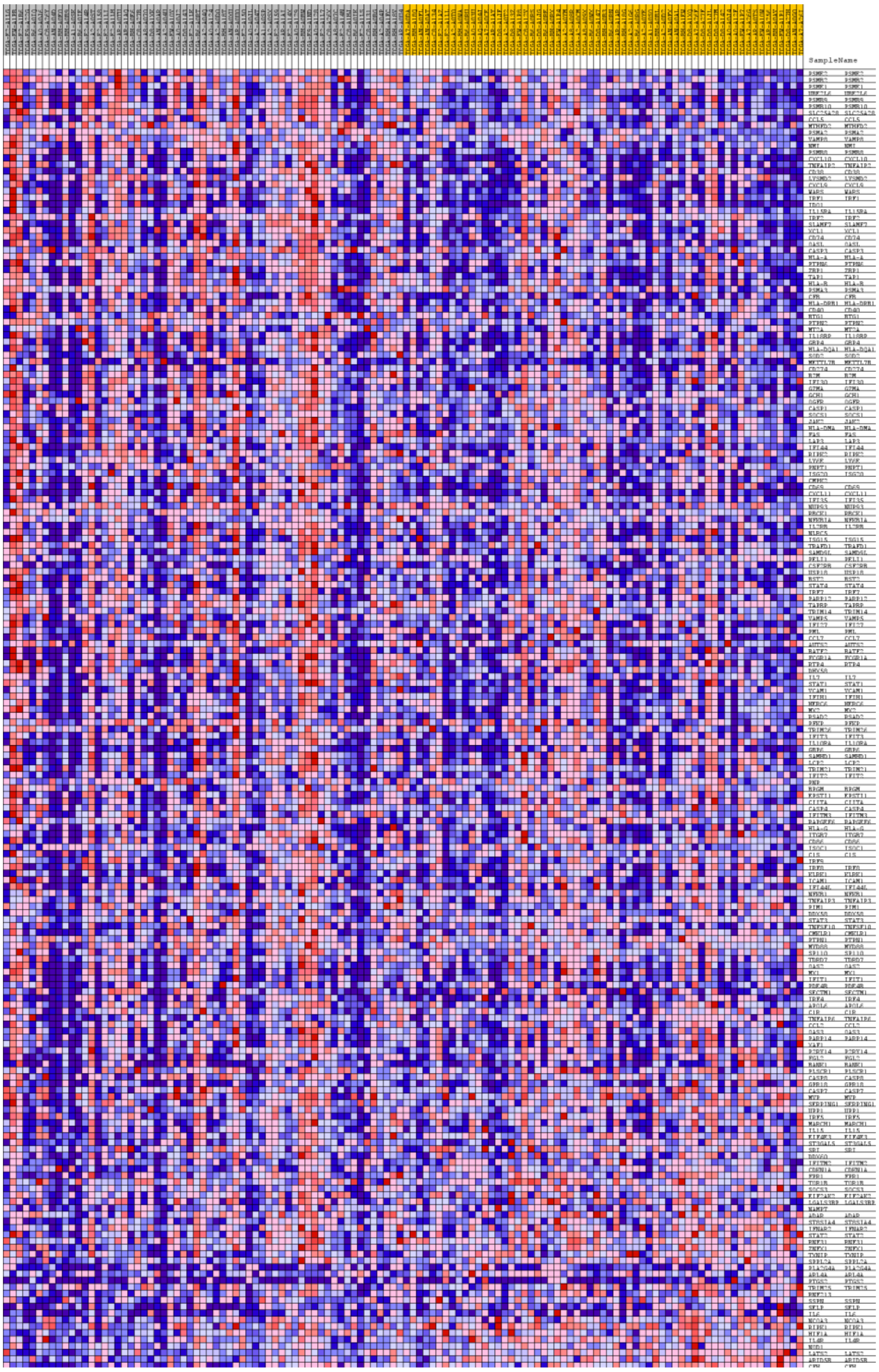

Figure S1: Gene Set Enrichment Analysis of tumor samples (TCGA breast cancer cohort). Heatmaps depicting gene signatures of hallmarks for epithelial mesenchymal transition (A), TGFβ signaling (B), lymphocyte activation (C) and IFNγ signature (D) in tumor samples expressing high (yellow) or low (grey) TSP1 levels (n=122).

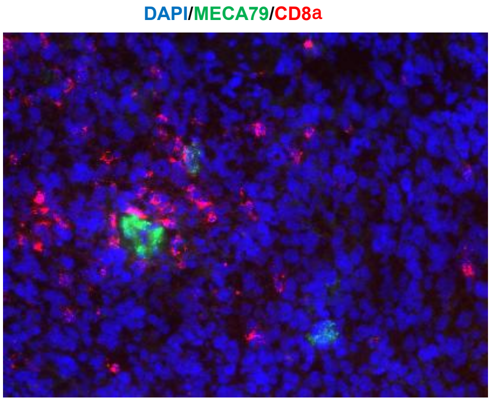

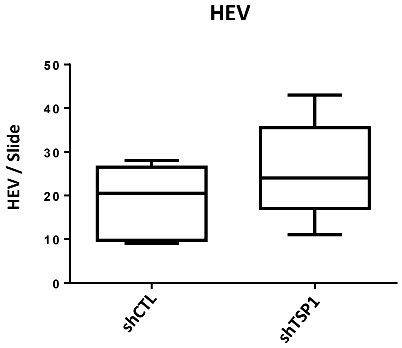

Figure S2: Impact of TSP1 on tumor high endothelial venules (HEV) in 4T1 mouse breast cancer model. Immunodetection of MECA79 and CD8α for the quantification of HEV cells in 4T1 shCTL and 4T1 shTSP1 tumors at day 11. Left panel, representative staining of 4T1 tumors. Right panel, values are numbers of HEV per slide counted on 6 tumors from each group. Data are represented as Tukey boxes.

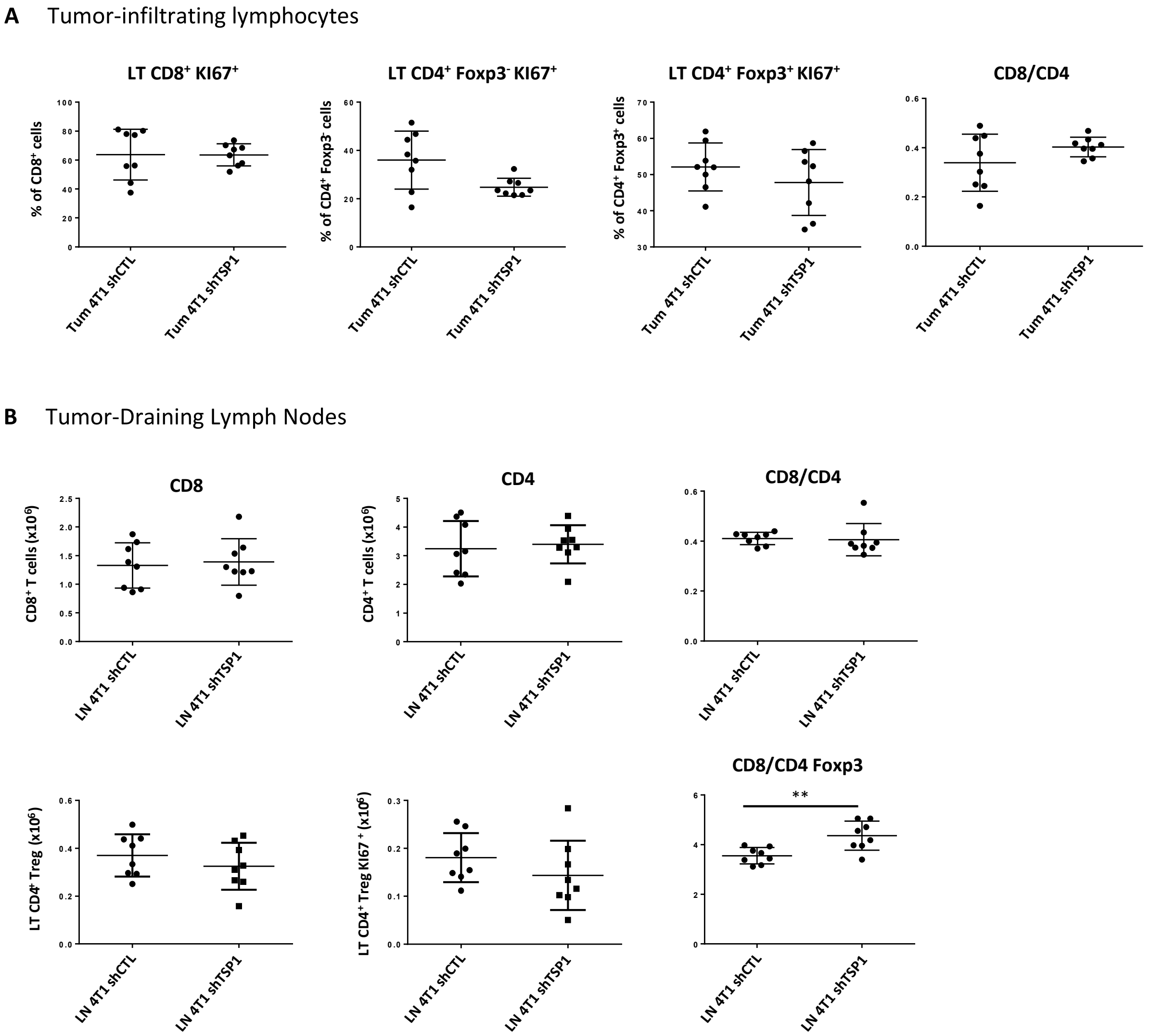

Figure S3: Impact of TSP1 on immune responses in 4T1 mouse breast cancer model. **A:** Flow cytometry results of KI67 positive expression in TILs. **B:** Analysis by flow cytometry of draining lymph node at day 11. Absolute numbers of CD8 T cells, CD4 T cells, Treg (CD4+ FOXP3+) and Treg KI67+ cells; right panels: CD8 to CD4 T cell ratio and CD8 T cell to Treg ratio. (**: p<0,01).

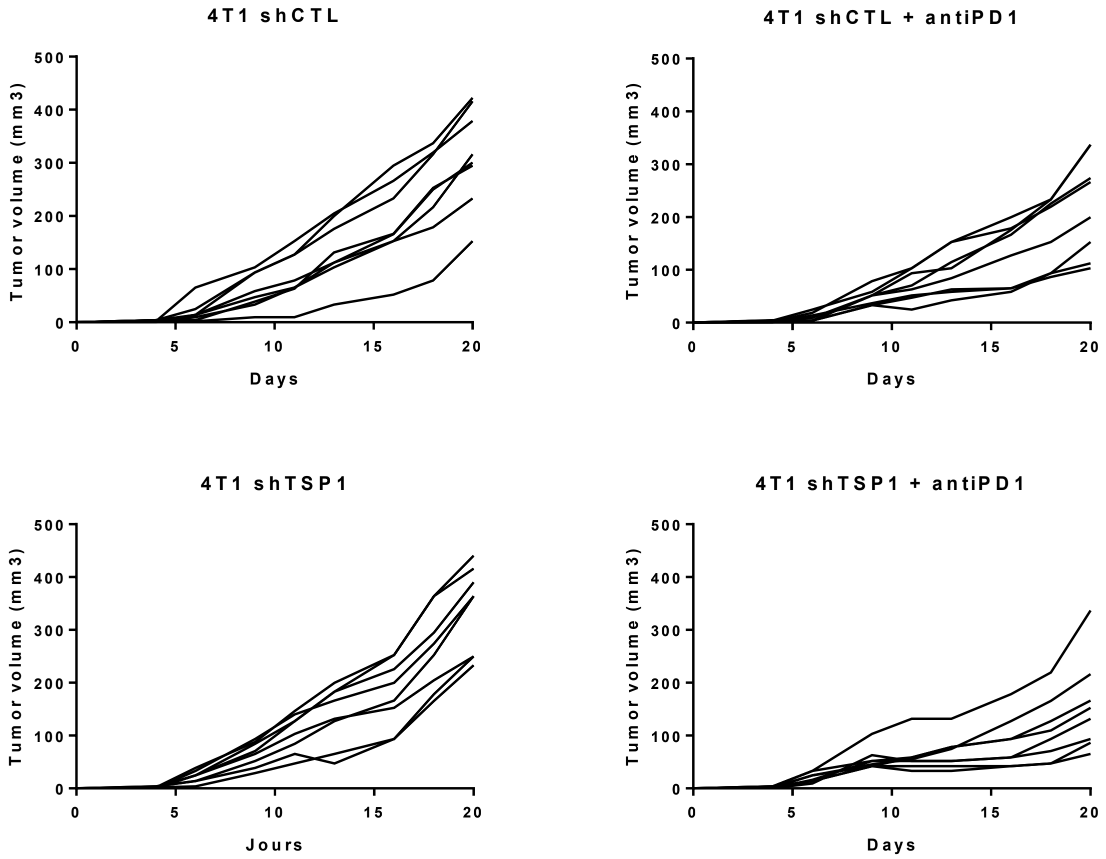

Figure S4: Impact of TSP1 on anti-PD-1 treatment in 4T1 mouse breast cancer model. Individual curves are for each tumor growth depicted in figure 5F.
